# The ability of energy recovery in professional soccer players is increased by individualized low-intensity exercise

**DOI:** 10.1371/journal.pone.0270484

**Published:** 2022-06-30

**Authors:** Jihwan Hwang, Na-Ram Moon, Oliver Heine, Woo-Hwi Yang

**Affiliations:** 1 Graduate School of Sports Medicine, CHA University, Seongnam-si, Gyeonggi-do, Republic of Korea; 2 Olympic Training Centre Rhineland, Cologne, North Rhine-Westphalia, Germany; 3 Department of Medicine, General Graduate School, CHA University, Seongnam-si, Gyeonggi-do, Republic of Korea; Universiti Malaya, MALAYSIA

## Abstract

The aim of this study was to investigate whether individualized low-intensity exercise (ILIE) within the recovery domain before lactate threshold 1 (LT 1) improves energetic recovery and general endurance capacity in professional soccer players. Twenty-four professional soccer players (age: 24.53 ± 4.85 years, height: 180 ± 6.30 cm, body mass: 75.86 ± 8.01 kg, body fat: 12.19 ± 2.69%) participated in the study (n = 24). The 1-h ILIE intervention involved 27 jogging sessions spanning nine weeks and jogging speed corresponding to 72% of LT 1 (7.15 ± 0.95 km∙h^−1^). Pre-ILIE and post-ILIE LT testing variables measured within 9 weeks included blood lactate concentrations (La^−^) and heart rate (HR) at specific exercise intensities during ILIE LT test. The jogging/running speeds (S), delta (Δ) S, HR, and ΔHR were measured at 1.5, 2.0, 3.0, and 4.0 mmol∙L^−1^ La^−^, respectively. Values of La^−^ and HR at the same exercise intensities (5.4–16.2 km∙h^−1^) in the post-ILIE LT test compared with pre-ILIE LT test were significantly decreased (*P* < 0.05 and *P* < 0.01, respectively). Furthermore, S at all specific La^−^ levels (1.5, 2.0, 3.0, and 4.0) were significantly increased, while HR at 2.0, 3.0, and 4.0 La^−^ decreased significantly (*P* < 0.05 and *P* < 0.01, respectively). Low to moderate positive correlations were observed between ΔS and ΔHR at 1.5 and 2.0 La^−^ (*r* = 0.52 and *r* = 0.40, respectively). The nine-week ILIE improved energy recovery and general endurance of professional soccer players. This relates to repeated high-intensity intermittent sprints during the 90-min soccer game.

## Introduction

Soccer play involves exercise at a wide range of intensities. High-intensity intermittent exercises (HIIE) such as running, sprinting, and cutting and low-intensity exercises (LIE) such as jogging, walking, and standing occur for different durations and intensities during the soccer play depending on several factors [1–3]. The technical level, style of play, tactical strategies, playing position, and the physiological capacity of individual players in both teams influence the work rate in the soccer match [2, 4]. Accordingly, the aerobic energy system is predominantly utilized. Aerobic energy is estimated at 90% of the energy expenditure, although the rate of anaerobic energy turnover is high during a 90-min soccer game [5, 6].

High-intensity movements such as repeated sprints and total distance in elite and sub-elite soccer players are gradually reduced in the second half compared with the first [3, 5, 7, 8]. Accordingly, the blood lactate levels were decreased, while the levels of plasma-free fatty acids increased in Danish soccer players from the first to second half of the game [5]. These findings indicate that carbohydrates such as muscle glycogen are mostly utilized in the first half, which impairs repeated high-intensity performances in the second half [5, 9]. However, increased energetic recovery is facilitated by adenosine triphosphate (ATP) re-synthesis from accumulated lactate and metabolic flexibility, reflecting the efficiency of fat and carbohydrate oxidation may contribute to successful performance in the 90-min soccer game [10–13].

HIIE such as repeated sprints with the ball requires increased phosphagen and glycolytic metabolism [14]. However, ATP is resynthesized rapidly from phosphocreatine (PCr) during LIE and the accumulated lactate via substrate-level and oxidative phosphorylation delays HIIE during the second half [5, 10, 11, 15, 16].

In this regard, lactate threshold (LT) tests have been used by sports scientists for more than fifty years to measure individualized exercise prescriptions for elite athletes. Furthermore, the blood lactate concentration under different exercise intensities is a sensitive biomarker of endurance than maximal oxygen uptake (VO_2max_) [10, 11, 17–21]. The improved recovery ability and general endurance are interpreted by the rightward shift of the exponential lactate curve in the LT test [10, 11, 17, 18, 22]. The blood lactate level in the LT test is used to measure different exercise intensities including zones 1 (low), 2 (threshold/moderate), and 3 (high) [10, 11, 23–25]. LIE at lactate levels < 2 mmol∙L^−1^ (zone 1) is separated into recovery and extensive exercises. The recovery and extensive exercises are defined before and until the first onset of the lactate curve (LT 1) [10, 11, 23, 26]. Most training volumes include predominantly zone 1 exercise intensities (≥ 87%) involving middle- and long-distance running such as marathons to improve athletes’ endurance [27]. Lee et al. [11] suggest that eight sessions of 1-h LIE for four weeks improve energy recovery during jogging (zone 1) and general endurance performance during running (zones 2 and 3) compared with 16 sessions of 30-min LIE for four weeks in adults. As well, this finding shows that 1-h LIE increases fat oxidation, ATP re-synthesis, and the efficiency of metabolic flexibility, resulting in improved energy recovery [10–12].

However, based on the aforementioned LIE as zone 1 (< 2 mmol∙L^−1^), it is not clear, whether individualized low-intensity exercise (ILIE) influences recovery and general endurance (rightward shift) in professional soccer players. Therefore, this study aimed to investigate whether the training intervention in recovery zone 1 (RZ 1) before LT 1 improved the energy recovery and general endurance in professional soccer players.

## Materials and methods

### Participants

A total of twenty-four professional soccer players participated in this study (n = 24). They were recruited from a team in the Korea Professional Football League (K League). The anthropometric parameters of the participants were as follows (mean ± standard deviation, SD): age, 24.53 ± 4.85 years; height, 180 ± 6.30 cm; body mass, 75.86 ± 8.01 kg; body fat, 12.19 ± 2.69%. Pre- and post-test data are presented in Table 1. The 24 participants included two forwards, five wingers, five midfielders, and twelve defenders. The players were involved in soccer-specific team training and resistance training (Table 2). The training volume was 13.5–16.5 hours per week during the preseason. The participants did not take any medication during pre- and post-test investigations and abstained from alcohol and nicotine consumption for at least 24 h before the experiment.

**Table 1:** Anthropometric data of pre- and post-test (n = 24).

Variables	Pre-test	Post-test
	(Mean ± SD)	(Mean ± SD)
Age [years]	24.53 ± 4.85	
Height [cm]	180 ± 6.30	
Body mass [kg]	75.86 ± 8.01	75.92 ± 8.06
Body fat [%]	12.19 ± 2.69	12.30 ± 2.58

**Table 2:** Regular training characteristics during the preseason.

	Soccer-specific team training	Resistance training
Training program	technical training	overhead squat, deadlift
	tactical training	dumbbell lunge, bench press
	small side game	overhead press, bent over row
	friendly match	lat pulldown, pull up
Hours per week	10.5–13.5	2 − 3

The study was approved by the Institutional Ethics Committee of CHA University (No. 1044308-202108-HR-065-01). The study protocols were in accordance with the Declaration of Helsinki. All participants signed an informed consent form.

### Study design

**Fig 1:**
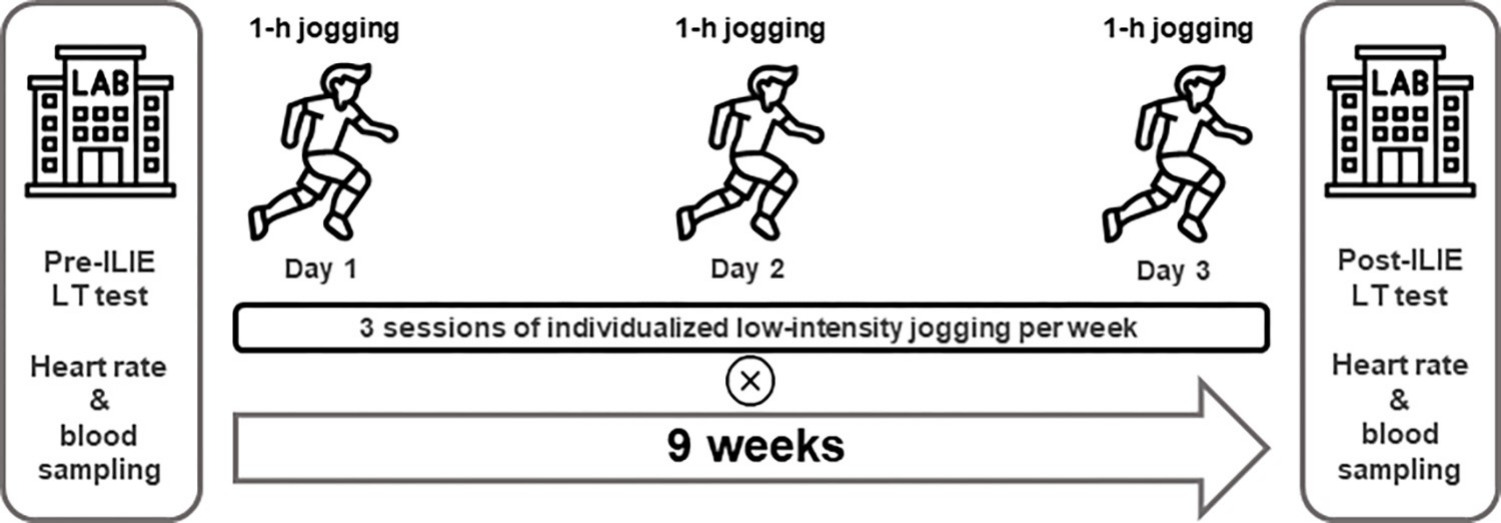
Study design and procedure. Pre-ILIE and post-ILIE LT tests were conducted over 9 weeks. All participants performed three individualized low-intensity jogging sessions per week according to the results of the pre-ILIE LT test. ILIE: individualized low-intensity exercise, LT: lactate threshold.

Pre-ILIE and post-ILIE LT tests were scheduled for 24 professional soccer players within nine weeks of the study (Fig 1). Test procedures were controlled under laboratory environmental conditions including a temperature of 23°C and a relative humidity of 50%. All participants were instructed not to change their diet during the exercise. Soccer-specific training, resistance training, and training volume per week remained unchanged if they were part of their usual training regimen. Furthermore, the participants’ nutritional intake was not considered during the study. However, no food intake was allowed for three hours before the measurements were performed [11, 28]. Participants completed the anthropometric measurement using an 8-electrode segmental multi−frequency bioelectrical impedance analysis (BIA, 50–1000 kHz) (InBody 770; InBody Co. Ltd., Seoul, Republic of Korea). The pre-ILIE and post-ILIE LT tests [11, 29, 30] were conducted on a treadmill (NR30XA, DRAX Corporation Ltd., Seoul, Republic of Korea).

### Laboratory LT testing

The pre-ILIE LT test was performed via graded incremental exercise on a treadmill with 5-min stages interspersed by 30s breaks between stages for La^−^ assessment. The initial jogging speed of 5.4 km∙h^−1^ (1.5 m∙s^−1^) was increased to 1.8 km∙h^−1^ (0.5 m∙s^−1^) every five minutes. The test was stopped when the blood lactate concentration (La^−^) was greater than 4 mmol∙L^−1^ after each running speed. The initial jogging stage was followed by an additional recovery (cooldown) stage (5 min) [11, 28–30]. The same set-up was utilized after nine weeks in the post-ILIE LT test. Jogging/running speeds and HR at 1.5, 2.0, 3.0, and 4.0 mmol∙L^−1^ La^−^ were calculated using a mathematical model of interpolation that has been reported previously [11, 30–32]. In addition, differences in jogging/running speed and HR at 1.5, 2.0, 3.0, and 4.0 mmol∙L^−1^ La^−^ between pre-ILIE and post-ILIE LT tests (ΔS and ΔHR at 1.5, 2.0, 3.0, and 4.0 La^−^, respectively) were calculated. To determine La^−^, capillary blood sampling was performed from the earlobe (20 μL) at rest and immediately after each stage. All La^−^ levels were analyzed using an enzymatic-amperometric sensor chip system (Biosen C line, EKF diagnostics sales GmbH, Barleben, Germany). The HR data were recorded using a Polar H10 sensor (Polar Electro, Kemple, Finland). The mean value of HR over the last 30 s of each stage was calculated for statistical analysis [11].

### Determination of individualized low-intensity exercises within recovery zone 1

**Fig 2:**
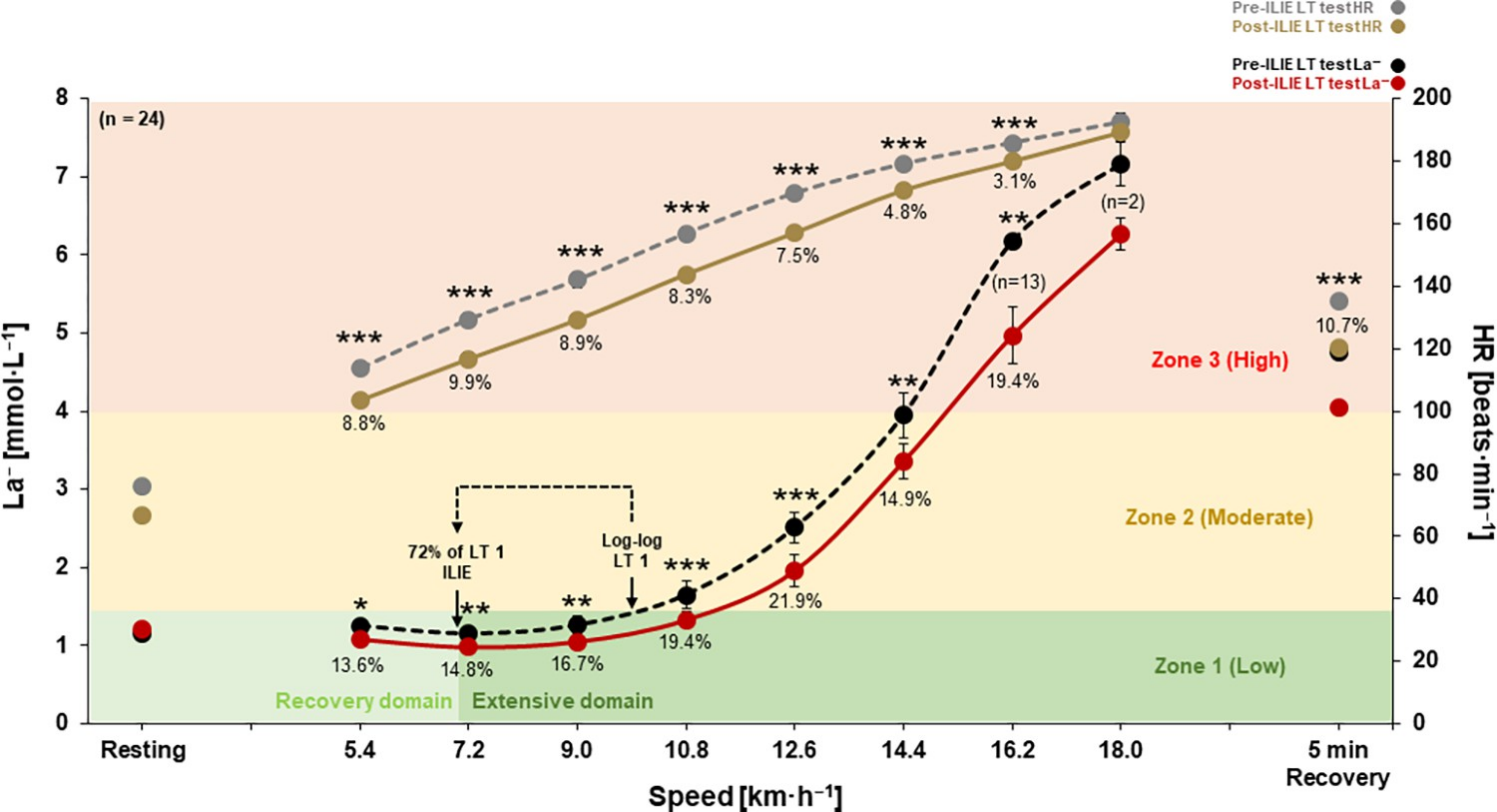
Outcomes of pre-ILIE and post-ILIE LT tests. La^−^: blood lactate concentrations, HR: heart rate, ILIE: individualized low-intensity exercise, %: percentages of improved La^−^ at certain jogging/running speeds between pre-ILIE and post-ILIE LT tests. *P < 0.05, **P < 0.01, and ***P < 0.001. Data are mean ± standard error of the mean (S.E.M).

During the usual training plan of professional soccer players, the nine weeks consisted of additional 27 jogging sessions of 1-h ILIE on the treadmill [10, 11, 28, 33]. ILIE was based on the results of pre-ILIE LT test in 72% of LT 1 (7.15 ± 0.95 km∙h^−1^) jogging speed within RZ 1 (recovery domain before the increased lactate curve within zone 1) [23, 26] (Table 3 and Fig 2). LT 1 was estimated using a mathematical model of log-log LT described previously [34, 35]. The percentage of LT 1 was suggested by our pilot study data, and the jogging speed < 70% of LT 1 was not appropriate for participants because it was too slow within RZ 1. Furthermore, the HR level was monitored during the 9-week ILIE intervention, which was digitally saved in the associated HR application [11, 28] (Table 3).

**Table 3:** Recommended individualized low-intensity exercise (jogging) speed.

Participants	Log-log LT 1	72% of log-log LT 1	Monitored 9-week HR_mean_ (beats∙min^−1^)
	speed (km∙h^−1^)	speed (km∙h^−1^)	
n = 24	(Mean ± SD)	(Mean ± SD)	(Mean ± SD)
	9.93 ± 1.32	7.15 ± 0.95	117 ± 11

LT: lactate threshold, HR_mean_: mean heart rate during 1-h jogging (9 weeks).

### Statistical analyses

All measured data were analyzed using GraphPad Prism 9.2 (GraphPad Prism Software Inc, La Jolla, CA, USA). Variables are presented as mean and standard deviation (SD)/standard error of the mean (S.E.M). The normal distribution of all variables was performed using the Shapiro-Wilk test. La^−^ and HR variables between pre-ILIE and post-ILIE LT tests (each stage) were compared using a paired *t*-test. At the stage of 16.2 km∙h^−1^, the results of only thirteen (n = 13) participants were statistically analyzed. At the stage of 18 km∙h^−1^, the results of two participants (n = 2) were not statistically analyzed. Jogging/running speed at 3.0 mmol∙L^−1^ La^−^ and HR at 3.0 and 4.0 mmol∙L^−1^ La^−^ between pre-ILIE and post-ILIE LT tests were also compared using a paired *t*-test. A Wilcoxon signed-rank test was used to compare jogging/running speeds at 1.5, 2.0, and 4.0 mmol∙L^−1^ La^−^ and HR 1.5 and 2.0 mmol∙L^−1^ La^−^ between pre-ILIE and post-ILIE LT tests. The alpha level of significance was set at *P* < 0.05 for all analyses. The effect sizes (ES; Cohen’s *d* and Z√N: *d* and *r*) were calculated for parametric and non-parametric tests. Thresholds for small, medium, and large effects were 0.2, 0.5, and 0.8 (parametric) and 0.1, 0.3, and 0.5 (non-parametric), respectively [36]. Furthermore, Spearman rank correlations were performed between ΔS and ΔHR at specific La^−^ concentrations.

## Results

### Comparison of La^−^ and HR between pre-ILIE and post-ILIE LT tests

In the post-ILIE LT test, values of La^−^ and HR from the first stage 5.4 km∙h^−1^ to 16.2 km∙h^−1^ were significantly decreased compared with the pre-ILIE LT test (5.4 km∙h^−1^: *P* = 0.017; ES [*d*]: 0.51, *P* < 0.001; ES [*d*]: 1.23, 7.2 km∙h^−1^: *P* = 0.004; ES [*d*]: 0.58, *P* < 0.001; ES [*d*]: 1.14, 9.0 km∙h^−1^: *P* = 0.003; ES [*d*]: 0.56, *P* < 0.001; ES [*d*]: 1.09, 10.8 km∙h^−1^: *P* < 0.001; ES [*d*]: 0.56, *P* < 0.001; ES [*d*]: 1.03, 12.6 km∙h^−1^: *P* < 0.001; ES [*d*]: 0.61, *P* < 0.001; ES [*d*]: 1.03, 14.4 km∙h^−1^: *P* = 0.003; ES [*d*]: 0.60, *P* < 0.001; ES [*d*]: 0.92, 16.2 km∙h^−1^: *P* = 0.009; ES [*d*]: 0.81, *P* < 0.001; ES [*d*]: 0.67, respectively). After 5-min recovery (cooldown) stage, only the HR was significantly decreased in the post-ILIE LT test compared with the pre-ILIE LT test (*P* < 0.001; ES [*d*]: 1.0). Altered percentages of each of the La^−^ and HR values between pre-ILIE and post-ILIE LT tests are presented in Fig 2.

### Comparisons of jogging/running speeds and HR at specific La^−^ between pre-ILIE and post-ILIE LT tests

**Fig 3:**
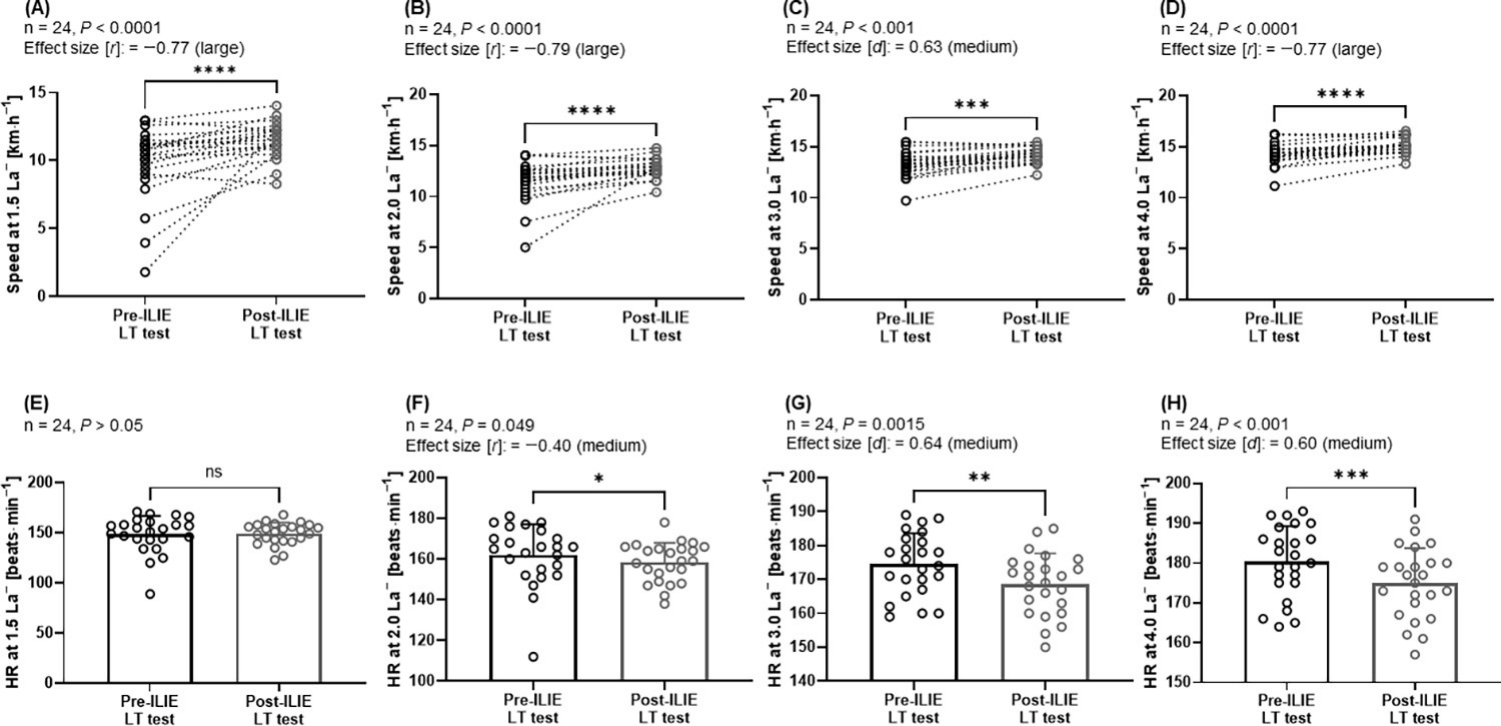
Jogging/running speeds and heart rate at certain blood lactate concentrations. (A–D) Jogging/running speeds (S) and (E–H) heart rate (HR) at specific blood lactate concentrations in mmol∙L^−1^ (La^−^ 1.5, 2.0, 3.0, and 4.0). ns: *P* > 0.05, *P < 0.05, **P < 0.01, ***P < 0.001, and ****P < 0.0001.

Jogging speeds at 1.5 and 2.0 mmol∙L^−1^ La^−^ in the post-ILIE LT test were significantly increased compared with the pre-ILIE LT test, and the HR at 2.0 mmol∙L^−1^ La^−^ in the post-ILIE LT test was significantly decreased compared with the pre-ILIE LT test (*P* < 0.0001; ES [*r*]: −0.77, *P* < 0.0001; ES [*r*]: −0.79, *P* = 0.049; ES [*r*]: −0.40, respectively) (Fig 3A, 3B and 3F). As well, the running speeds at 3.0 and 4.0 mmol∙L^−1^ La^−^ in the post-ILIE LT test were significantly increased compared with the pre-ILIE LT test, while the HR values were significantly decreased (*P* < 0.001; ES [*d*]: 0.63, *P* < 0.0001; ES [*r*]: −0.77, *P* = 0.0015; ES [*d*]: 0.64, *P* < 0.001; ES [*d*]: 0.60, respectively) (Fig 3C, 3D, 3G and 3H).

### Correlations between ΔS and ΔHR at different La^−^concentrations

**Fig 4:**
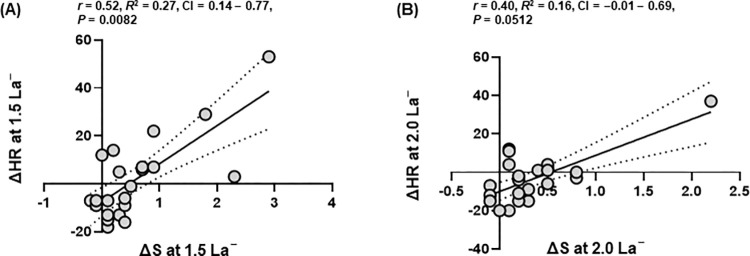
Correlation analyses. (A) Spearman rank correlation between delta jogging speed (ΔS) at 1.5 mmol∙L^−1^ blood lactate concentration (La^−^) and delta heart rate (ΔHR) at 1.5 mmol∙L^−1^ La^−^ (B) Spearman rank correlation between ΔS at 2.0 mmol∙L^−1^ La^−^ and ΔHR at 2.0 mmol∙L^−1^ La^−^.

Values of ΔS and ΔHR at specific La^−^ concentrations are presented in Table 4 and Fig 4A and 4B indicate the low to moderate positive correlations between ΔS and ΔHR at 1.5 and 2.0 mmol∙L^−1^ La^−^ (correlation coefficient [*r*] = 0.52, *R*^*2*^ = 0.27, 95% confidence interval [95% CI] = 0.14–0.77, *P* = 0.0082, *r* = 0.40, *R*^*2*^ = 0.16, 95% CI = –0.01–0.96, *P* = 0.0512, respectively).

**Table 4:** Differences of jogging/running speeds and heart rate between pre-ILIE and post-ILIE LT tests (n = 24).

Specific La^−^	ΔS (km∙h^−1^)	ΔHR (beats∙min^−1^)
	(Mean ± SD)	(Mean ± SD)
1.5 mmol∙L^−1^	2.01 ± 2.74	1.38 ± 16.41
2.0 mmol∙L^−1^	1.23 ± 1.68	−3.58 ± 12.41
3.0 mmol∙L^−1^	0.80 ± 0.88	−5.88 ± 7.99
4.0 mmol∙L^−1^	0.74 ± 0.75	−5.46 ± 6.90

ΔS: difference of jogging/running speed between pre-ILIE and post-ILIE LT tests, ΔHR: difference of heart rate between pre-ILIE and post-ILIE LT tests.

## Discussion

Exercise intensity levels in LIE performed at La^−^ concentrations below 2 mmol∙L^−1^ affecting energetic recovery and general endurance performance (rightward shift) in professional soccer players remain unknown. To the best of our knowledge, this is the first study that identifies the recovery training zone 1 (focused on jogging speed) precisely and its effects on the exponential lactate curve, energetic recovery ability, and general endurance of professional soccer players.

Major findings of this study suggest that a 9-week 1-h ILIE (72% of log-log LT) in professional soccer players can reduce La^−^ levels and HRs at similar exercise intensities (5.4–16.2 km∙h^−1^) in the post-ILIE LT test. The entire lactate curve indicated improvements for zones 1, 2, and 3 (rightward shift) between pre-ILIE and post-ILIE LT tests (Fig 2). Further, jogging/running speeds at 1.5, 2.0, 3.0, and 4.0 mmol∙L^−1^ La^−^ were increased in the post-ILIE test, whereas HR at 2.0, 3.0, and 4.0 mmol∙L^−1^ La^−^ decreased (Fig 3). These improvements at specific La^−^ might be related to total increased training volume during the preseason. In previous studies, an observational period of up to eight weeks within the preseason in Italian professional male soccer players was associated with an increased submaximal aerobic fitness such as running speed at 2 and 4 mmol∙L^−1^ La^−^ as well as zone 2 [37, 38].

However, usual soccer-specific training appears to have no ability to improve the entire exponential lactate curve including the recovery domain of professional soccer players based on previous studies [10, 11, 25, 28].

A previous study showed that ILIE (< 2.0 mmol∙L^−1^) of at least 1-h, twice a week, for 4 weeks improved jogging/running speed of actively trained adults at certain La^−^ levels [11]. Positive effects of LIE and polarized training lasted over nine weeks in athletes [25, 28]. This was also confirmed in our study.

During certain stages of the LT test, decreased La^−^ levels can be explained by the increased use of total fat as an energy source in ILIE [11, 39–42]. This phenomenon explains the increase in jogging/running speeds at certain La^−^ levels in this study [10, 11, 26, 42]. Furthermore, fat and La^−^ metabolism are largely related to mitochondrial abundance and function. LIE of 25% VO_2max_ mainly induces utilization of plasma fatty acids to produce energy, whereas the production of lactate is reduced for ATP re-synthesis [10, 11, 43–45]. In this regard, more pyruvate and lactate are utilized aerobically than are generated by anaerobic glycolysis [10, 46]. Indeed, a rightward shift of the exponential lactate curve can be indirectly explained by improved metabolic flexibility reflecting the efficiency of fat and carbohydrate oxidation, mitochondrial function, and oxidative capacity [11, 12].

Jogging speed at specific La^−^ recovery domain was specifically recommended for professional soccer players because percentages of estimated HR_max_ and VO_2max_ were not sensitive variables during their ILIE sessions [10, 17–21]. Furthermore, low to moderate positive correlations between ΔS and ΔHR at 1.5 and 2.0 mmol∙L^−1^ La^−^ could explain the jogging speeds within RZ 1 under the recommended exercise intervention. The suggested ILIE intensity at 72% LT 1 strongly induces gluconeogenesis such as the Cori cycle. During LIE, lactate is predominantly transported via blood from muscle cells to liver/kidney (Cori cycle) [47]. This mechanism is supported by increased hepatic blood flow with similar values of hepatic lactate uptake (0.55 ± 0.25 mmol∙min^−1^) and muscle lactate output (0.5 ± 0.3 mmol∙min^−1^). In contrast, diminished lactate synthesis occurs in muscle cells via an intracellular lactate shuttle mechanism during LIE [10, 11, 47]. These mechanisms of improved energy recovery support improving high-intensity intermittent actions during a 90-min soccer play [10, 11, 16, 45, 48, 49].

The 9-week ILIE decreased HR during the post-ILIE LT test. Dimensions of the left ventricular diastolic cavity, including mass, wall thickness, and stroke volume can be increased by long-term or long-distance training [50–52]. LIE including high-volume training causes adaptive alterations in cardiovascular function. The heart increases its ability to pump blood which is mainly influenced by increased stroke volume due to an increase in left ventricular mass. Generally, endurance training can reduce metabolic stress on the heart at rest and under submaximal exercise intensity [51].

Furthermore, reduced HR values after 9-week ILIE might be related to altered cardiac sympathetic and parasympathetic modulation [11, 53–55]. In particular, heart rate variability and parasympathetic activation might be increased after three sessions of ILIE for 9 weeks [11, 56]. However, these results should be analyzed along with additional HR-related variables such as blood pressure, HR_max_, and heart variability.

The current study has some limitations. No control group was used in this study because of training camp schedules of other professional soccer teams during the preseason. In addition, other metabolic-related variables such as fat and carbohydrate oxidation (g∙min^−1^) based on stoichiometry, respiratory exchange ratio, and VO_2_ data were not analyzed. Further studies are needed to address these limitations.

Our findings revealed that a jogging intensity of 72% from log-log LT 1, in which professional soccer players performed three sessions each week for 9 weeks, also suggested an improvement of fat oxidation and ATP re-synthesis in addition to general endurance in zones 1, 2, and 3. Repeated powerful sprints are highly dependent on anaerobic energy pathways (phosphagen and glycolytic energy systems) and can be increased by soccer-specific high-intensity interval training [57]. However, soccer players and trainers should additionally consider individualized low-intensity exercise sessions with a focus on re-metabolized energy from lactate to improve soccer players’ recovery ability. This ability as an aerobic base can lead to greater volumes of repeated high-intensity intermittent sprints during a soccer match [48, 58].

## Conclusions

Outcomes of the current study indicate that 9-week ILIE within RZ 1 can enhance energy recovery and general endurance of professional soccer players. ILIE focuses on the jogging speed of 72% from LT 1 (no percentage of estimated HR_max_). Efficient fat oxidation during LIE is important for aerobic conditioning between the soccer play and high-intensity intermittent sprints. Therefore, professional soccer players require an additional 1-h of ILIE for replenishing ATP levels and optimize the repeated high-intensity intermittent actions during the 90-min soccer play.
